# The use of XLSTAT in conducting principal component analysis (PCA) when evaluating the relationships between sensory and quality attributes in grilled foods

**DOI:** 10.1016/j.mex.2020.100835

**Published:** 2020-02-22

**Authors:** Natalia P. Vidal, Charles F. Manful, Thu H. Pham, Peter Stewart, Dwayne Keough, RaymondH. Thomas

**Affiliations:** a School of Science and the Environment/Boreal Ecosystem Research Initiative, Grenfell Campus, Memorial University of Newfoundland, Corner Brook A2H 5G4, Canada; b Algoma University, 1520 Queen St E, Sault Ste. Marie, ON P6A 2G4, Canada

**Keywords:** Grilled ruminant meat, Volatile metabolites, Unfiltered beer-based marinades, SPME-GC/MS, Sensory analysis, Principal component analysis

## Abstract

Multivariate statistics is a tool for examining the relationship of multiple variables simultaneously. Principal component analysis (PCA) is an unsupervised multivariate analysis technique that simplifies the complexity of data by transforming them in a few dimensions showing their trends and correlations. Interests in XLSTAT as statistical software program of choice for routine multivariate statistics has been growing due in part to its compatibility with Microsoft Excel data format. As a case of study, multivariate analysis is used to study the effects of unfiltered beer-based marination on the volatile terpenes and thiols, and sensory attributes of grilled ruminant meats. PCA was conducted to determine the correlations between the abundances of volatile terpenes and thiols and sensory attribute scores in marinated grilled meats, as well as to analyze if there was any clustering based on the type of meat and marination treatments employed.

• XLSTAT PCA output successfully reduced the number of variables into 2 components that explained 90.47% of the total variation of the data set.
• PCA clustered marinated and unmarinated meats based on the presence and abundances of volatile terpenes, thiols and consumer sensory attribute scores.
• PCA could be applied to explore relationships between volatile compounds and sensory attributes in different food systems.

## Graphical abstract

**Figure:**
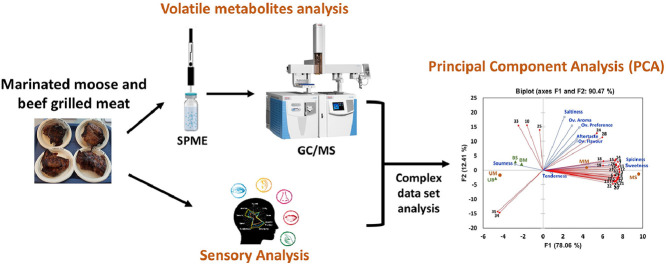

**Table:** Specification table

Subject Area:	Chemistry
More specific subject area:	Food Science
	Grilled Food Systems
	Food Quality
Method name:	The use of XLSTAT in conducting principal component analysis (PCA) when evaluating the relationships between sensory and quality attributes in grilled foods
Name and reference of original method:	[1] Vidal, Natalia P., et al. “Novel unfiltered beer-based marinades to improve the nutritional quality, safety, and sensory perception of grilled ruminant meats” Food chemistry 302 (2020): 125326.
	[2] Addinsoft (2019). XLSTAT statistical and data analysis solution. Long Island, NY, USA. https://www.xlstat.com.
Resource availability:	[1] https://help.xlstat.com/customer/en/portal/topics/824676-analyzing-data-1-2-?b_id=9283
	[2] https://help.xlstat.com/customer/en/portal/topics/824677-modeling-data-1-3-?b_id=9283
	[3] https://help.xlstat.com/customer/en/portal/topics/824671-correlation-association-tests?b_id=9283
	[4] https://help.xlstat.com/customer/en/portal/topics/824682-multiblock-data-analysis/questions?b_id=9201

## Method details

### Rational

Meat is an excellent source of nutrients including proteins, dietary fatty acids, essential minerals, and vitamins. The nutritional and sensory quality (e.g., appearance, texture, aroma, and flavor) are 2 key factors which determine consumers meat choice [1]. Meat marination is the process of incubating the meat into a seasoned liquid base before cooking. This process adds new compounds, which could have antioxidant properties and flavours, improving the sensory characteristics and preserving the meat nutritional quality. In a previous study, moose and beef steaks were marinated with two novel formulations of unfiltered beer-based marinades, and grilled [2]. The volatile profile and sensory test analyses of the grilled meat were complex data sets (more than 100 volatile compounds were identified, and 9 sensory attributes were scored in each sample) requiring the use of multivariate statistics for their analysis. Principal component analysis (PCA) is a multivariate statistical technique applied to reduce the number of variables (i.e., volatile metabolites) into a few uncorrelated variables named principal components (or factors) based on patterns of correlation of the original variables [3,4]. XLSTAT is a statistical software that can be employed to perform multivariate analysis of complex data sets. The aim of this MethodsX paper is to present a detailed step-by-step data analysis approach to demonstrate the use of principal component analysis to summarize, visualize and interpret the volatile metabolites and sensory attributes of marinated and unmarinated grilled ruminant meat using XLSTAT as the platform.

### Samples preparation

Detailed procedures for marinade formulation and composition, beef and moose meat marination and grilling conditions are provided in our previous publication [1]. Briefly, two ruminant meat types (beef, B, and moose, M,) were marinated with two unfiltered beer-based marinades (S, M). Control samples were left unmarinated (BU, MU). After grilling, one gram of ground meat of each sample was weighed and placed in a glass vial. The extraction of the volatile metabolites was performed by Solid Phase Microextraction and the profile analyzed by Gas Chromatography/Mass Spectrometry. Extraction procedure, analytical instrument conditions, as well as the volatile metabolites identification and semi-quantification procedures are detailed in [1].

### Consumer sensory evaluation

A sensory consumer test was performed on the unmarinated and marinated grilled moose and beef samples. Memorial University of Newfoundland (MUN), Grenfell Campus Research Ethics Board approved the procedures for use of human subjects for the sensory panel evaluations. The attributes assessed were sweetness, saltiness, sourness, spiciness, aftertaste, tenderness, overall flavor, overall aroma, and overall preference. Each sample was evaluated on a 10 cm line below the question, with 0 meaning low desirability/ or low flavor intensity and 10 meaning high desirability/ or high flavor intensity. Participants (*n* = 121) were instructed to place a mark through the line.

### Statistical analysis

A multivariate analysis approach was applied to the volatile metabolites detected using XLSTAT (Addinsoft, New York, USA). Principal Component Analysis (PCA) was performed on the abundances of the volatile compounds detected in the headspace and the sensory attributes scores of the samples to differentiate the unmarinated and marinated grilled beef and moose samples and to analyze possible relationships between them. In addition, one-way analysis of variance (ANOVA) was used to determine if there were significant differences between the volatile compounds observed in marinated and unmarinated moose and beef samples. Where treatment effects were significant, the means were compared with Fisher's Least Significant Difference (LSD), α = 0.05. To determine the linear correlations of volatile compounds and consumer sensory perceptions of the meat samples, Pearson's correlation coefficients were used. Figures were prepared using XLSTAT (Addinsoft, New York, USA).

### Principal component analysis

**Fig. 1:**
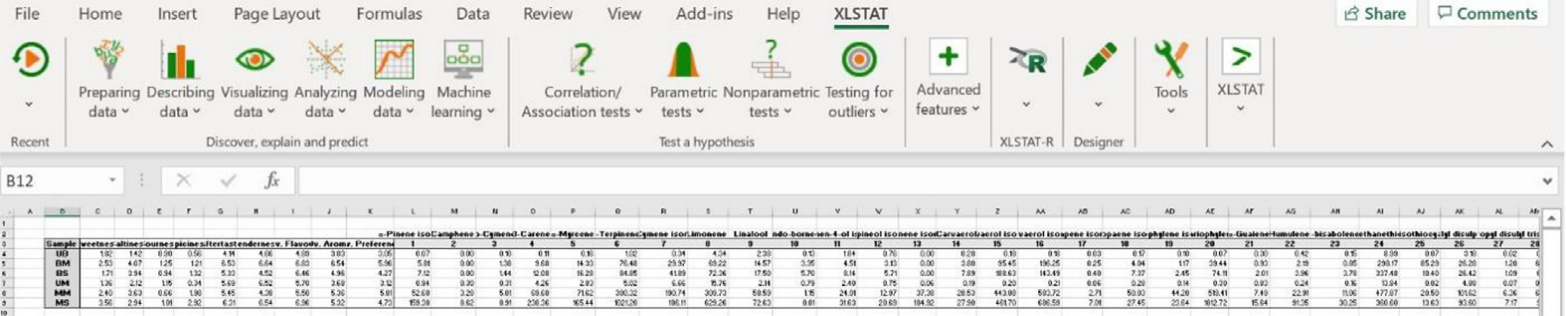
Principal component analysis (PCA) set up in XLSTAT to evaluate the relationships between the abundances of volatile terpenes and thiols detected in the headspace of unmarinated and marinated grilled beef and moose, and their sensory attributes scored by consumer panelists.

**Fig. 2:**
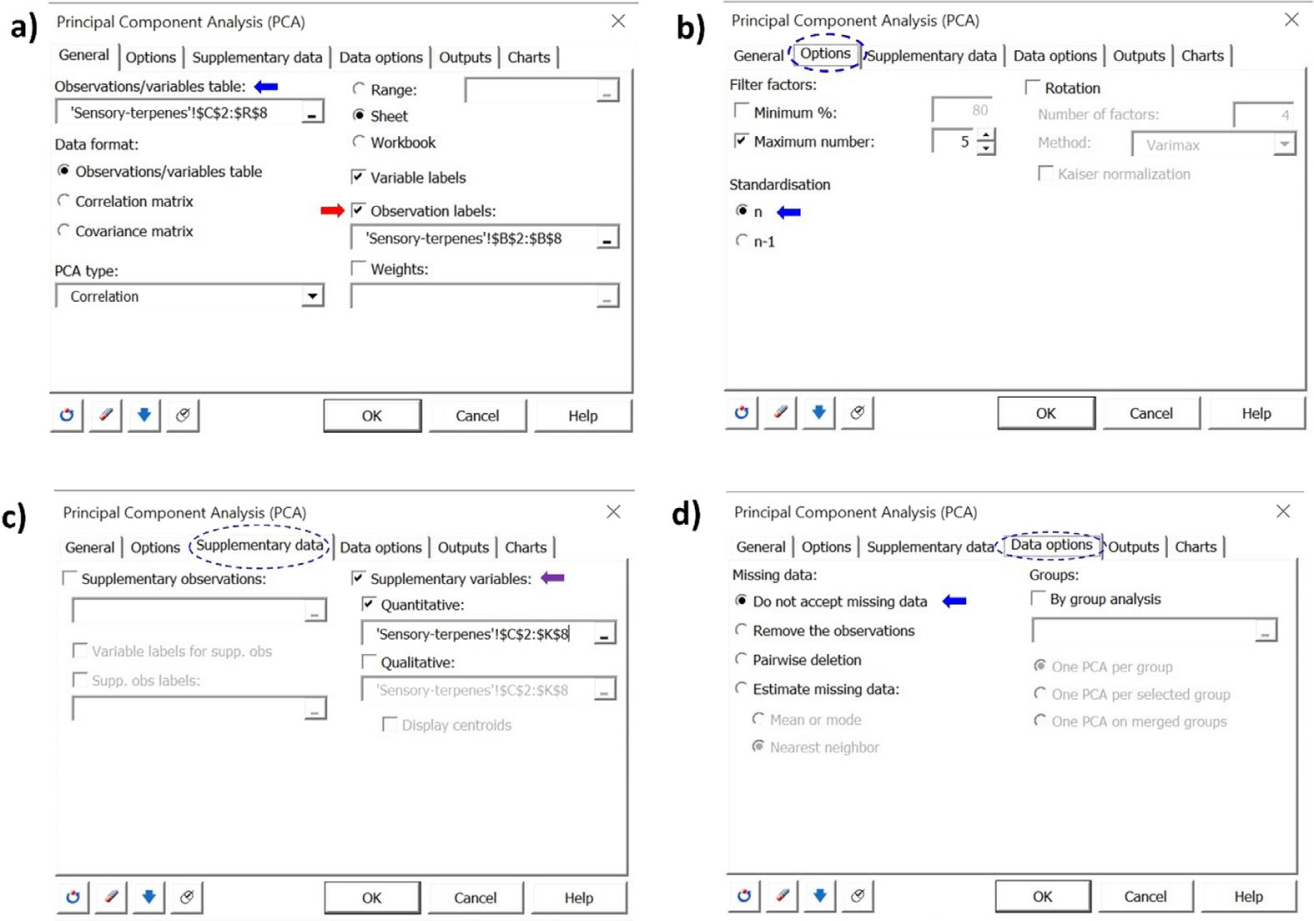
PCA set up for the analysis of the volatile metabolites abundances and sensory attributes score data sets in marinated and unmarinated grilled ruminant meat.

**Fig. 3:**
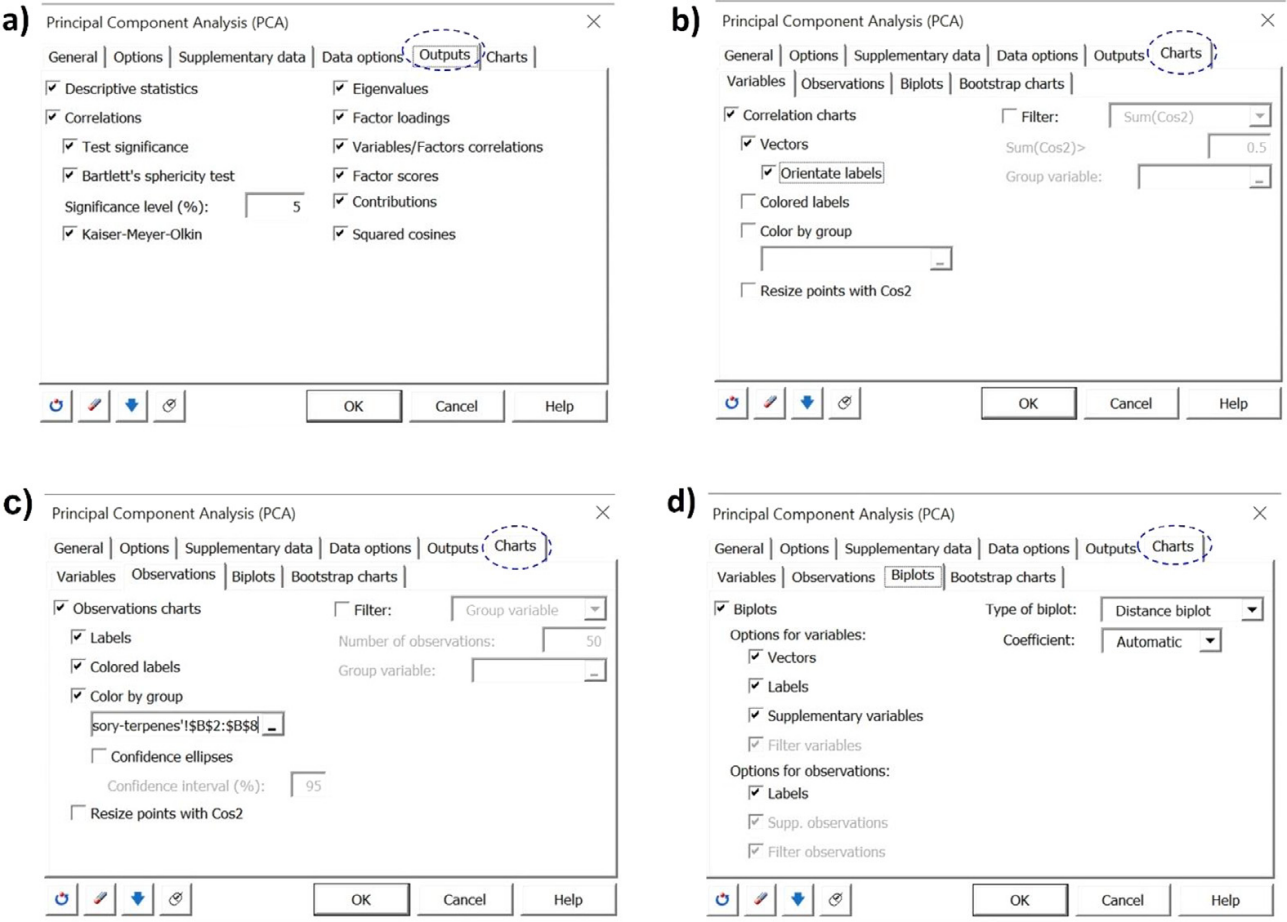
PCA set up for the analysis of the volatile metabolites abundances and sensory attributes score data sets in marinated and unmarinated grilled ruminant meat.

**Fig. 4:**
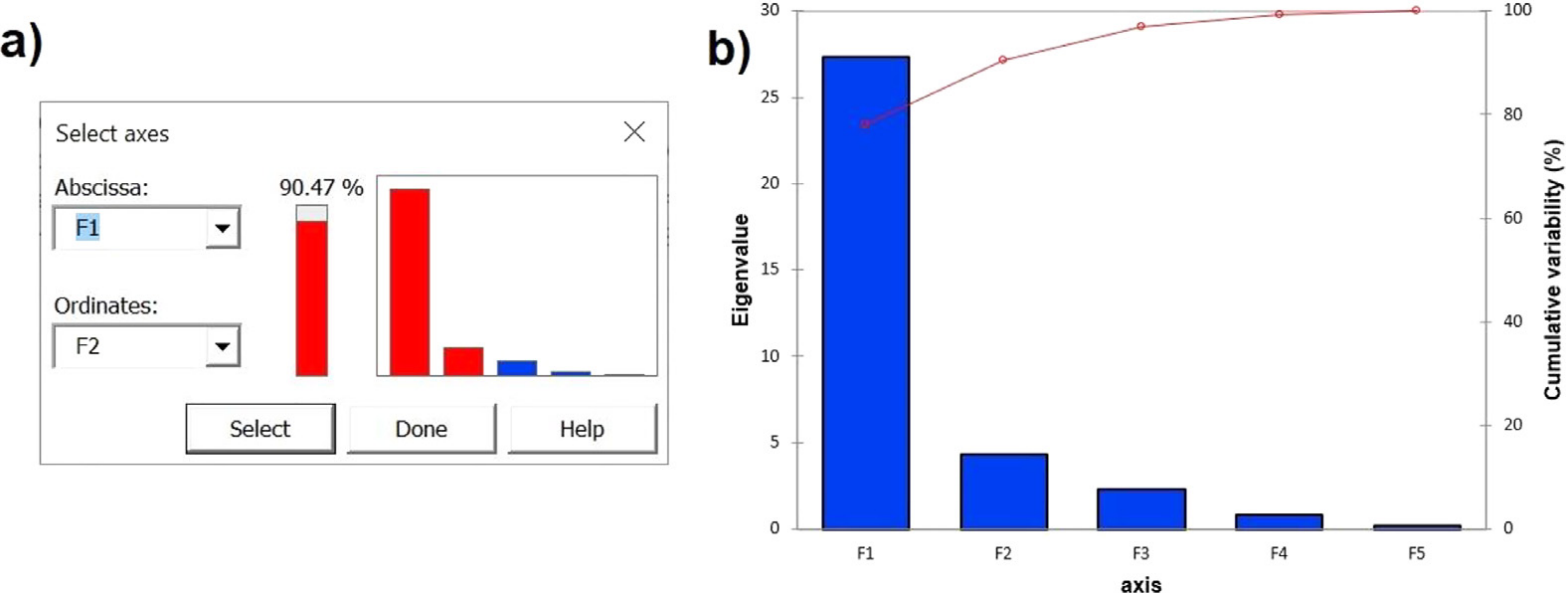
(a) Total variance explained (90.47%) in factors or principal component 1 and 2 of the PCA. (b) Total variance explained with the first 5 components of the PCA.

The volatile metabolites present in the headspace of the unmarinated and marinated grilled ruminant meats were identified and subsequently semi-quantified based on the area counts × 10^−6^ of the base peak. The consumer evaluation of the sweetness, saltiness, sourness, spiciness, aftertaste, tenderness, overall flavor, overall aroma, and overall preference of the unmarinated and marinated grilled meat samples was also performed. A data set consisting of a total of 35 volatiles (23 terpenes and 12 thiol compounds) and 9 consumer sensory attributes in each sample are considered in this study. This corresponds to 3591 data points (3 experimental treatments x 35 volatiles x 3 replicates = 315 data points; 3 experimental treatments x 9 sensory attributes x 121 consumer panelists = 3267 data points) for each meat type, or 7164 data points for beef and moose merged data. A statistical analysis of this data set based on either univariate descriptive or explorative methods to determine how marination affects the presence and abundance of volatiles and sensory attributes of meat samples, as well as the relationship between both data sets, will be tedious, computationally tasking and inefficient given the large data set under consideration. Multivariate exploratory methods such as principal component analysis (PCA), redundancy analysis (RDA), and hierarchical cluster analysis (HCA) were considered, of which PCA was found to be best suited owing to its simplicity, interpretation quality and usefulness to explaining the variation in our data set when conducted with XLSTAT statistical software. A step-by-step procedure for conducting PCA in XLSTAT to evaluate the effect of marination on the presence and abundance of volatile terpenes and thiols, as well as on sensory attributes (sweetness, saltiness, sourness, spiciness, aftertaste, tenderness, overall flavor, overall aroma, and overall preference) and their relationships in this kind of data set (grilled ruminant food) is shown in Fig. 1, Fig. 2, Fig. 3, Fig. 4. Procedure is as follows:

Step1: Start XLSTAT command to commence using XLSTAT

Step2: Select Analyzing data/ Principal components analysis command

Step3: Select data on the Excel sheet in the principal component dialog box. The Data format chosen is observations/ variables because of the format of the input data. The PCA standardization used during the computations is based on Pearson's correlation. (Fig. 2a)

Step 4: In Options tab → standardize the data by checking activate “n” standardization (Fig. 2b)

Step 5: Quantitative supplementary variables (sensory attributes scores) are included in this study. Thus, click in supplementary data tab → activate Supplementary variables and Quantitative boxes to select the consumer sensory attribute scores as supplementary quantitative variables. It is important to mention that quantitative supplementary variables have no effect in calculating the distance between individuals in the PCA plot. They will assist in the interpretation of the results. (Fig. 2c)

Step 6: Proceed to Data options tab → select “Do not accept missing data” for missing data. (Fig. 2d).

Step 7: In the Outputs tab → activate the options for Descriptive statistics, which will give a summary table with the descriptive statistics of our data set, and Correlations including Test Significance, Bartlett's sphericity test (significance level of 95%) which will test the hypothesis that your correlation matrix is an identity matrix, and Kaiser-Meyer-Olkin which will test the suitability of our data set for factor analysis. (Fig. 3a)

Steps 8: In the Charts tab → variables sub tab, check boxes for Correlations charts and Vectors to display these outputs. (Fig. 3b)

Step 9: In the Charts → Observations sub tab, check the boxes for Observation charts, Colored labels, Color by group in order to display the labels observations in color. Based on the selection made for the observation labels, the observations are selected to be colored by group in PCA map. (Fig. 3c)

Step 10: Proceed to Charts → Biplots sub tab, check boxes for Biplots. Under Options for variables check boxes for Vectors and Labels. For Options for Observations, check box for Labels. Set Type of biplot and Coefficient to Distance biplot and Automatic respectively. (Fig. 3d)

Step 11: Proceed to Charts → Bootstrap charts sub tab and uncheck the Bootstrap observations chart option. Click OK to start PCA computations based on data selections and configurations made.

Step 12: Select the principal component for which you want to display the plots. For this data set, the sum of the first two factors accounts for 90.47% of the total variation in the data. Click Done to output PCA results.

### Case of study: PCA performed on the volatile compounds abundances and sensory attributes scores of grilled beef and moose meats

**Fig. 5:**
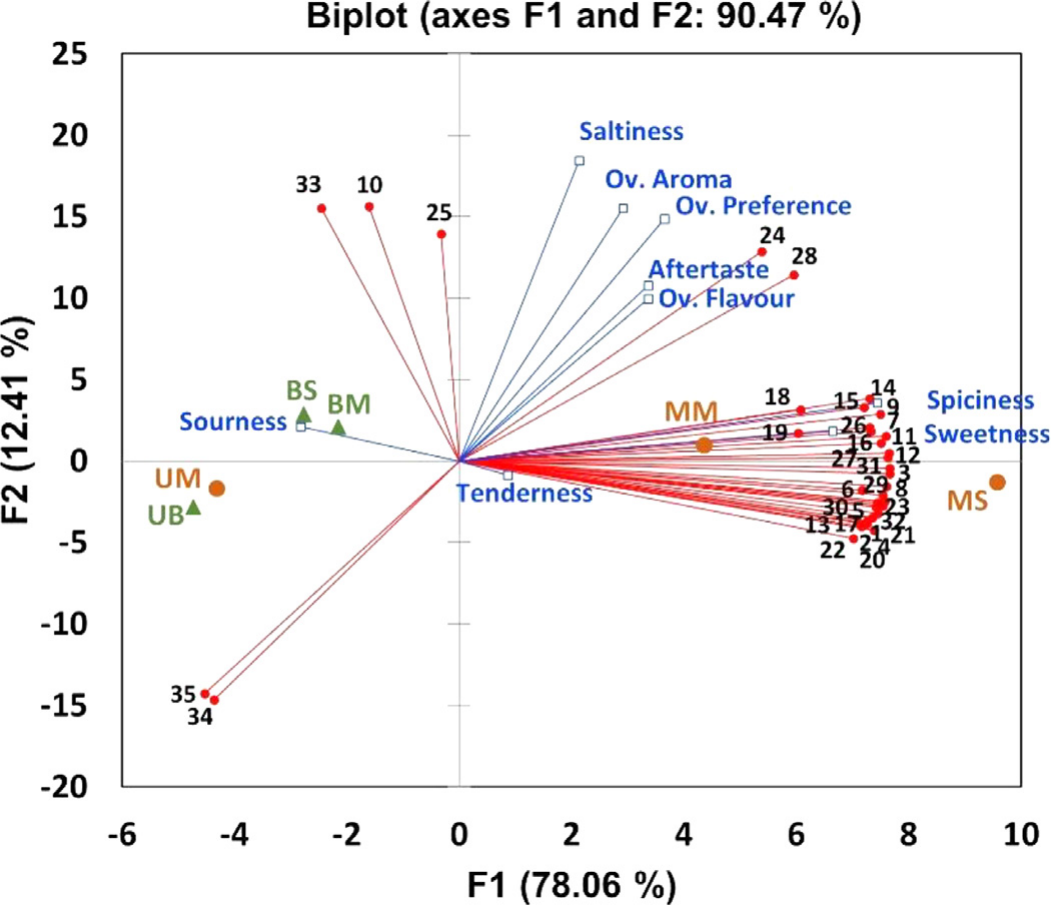
PCA biplot performed with the abundances of the volatile terpenes and thiols detected in the headspace of unmarinated and marinated grilled moose and beef, together with the scores of sensory attributes as supplementary quantitative variables. UM: unmarinated moose, UB: unmarinated beef, MM: marinated moose with unfiltered beer based marinate 1, MS: marinated moose with unfiltered beer based marinate 2, BM: marinated beef with unfiltered beer based marinate 1; BS: marinated beef with unfiltered beer based marinate 2 [1]. 1: α-Pinene isomer; 2: Camphene; 3: o-Cymene; 4: 3-Carene; 5: α-Myrcene; 6: α-Terpinene; 7: o-Cymene isomer; 8: Limonene; 9: Linalool; 10: Endo-borneol; 11: Terpinen-4-ol isomer; 12: Terpineol isomer; 13: Elemene isomer; 14: Carvacrol; 15: Carvacrol isomer; 16: Carvacrol isomer; 17: Coapene isomer; 18: α-Copaene isomer; 19: Cariophylene isomer; 20: Cariophylene; 21: α-Guaiene; 22: Humulene; 23: α-Bisabolene; 24: Methanethiol; 25: Allyl isothiocyanate; 26: Diallyl disulphide; 27: Dipropyl disulfide; 28: Allyl trisulfide; 29: Diallyl sulfide; 30: 2-Ethyl dithiane; 31: Allyl-2-isopropyldisulfane; 32: (E)−1-(Prop-1-en-yl)−3-propyltrisulfane isomer; 33: 3,4-Dimethyl-thioene; 34: Dimethyl-disulfide; 35: Dimethyl trisulfide.

Principal component analysis (PCA) was conducted using XLSTAT to explore relationships between sensory attributes and volatile compounds detected in the headspace of marinated and unmarinated moose and beef samples. A detailed step-by-step guide to setting up XLTATS to run PCA was shown in Fig. 1, Fig. 2, Fig. 3, Fig. 4. After testing that Bartlett's sphericity test was < 0.05 and KMO values were 0.648 (acceptable value), it can be seen that grilled beef and moose samples grouped in distinct quadrants of PCA biplot based on the abundances of volatiles and sensory attribute scores for the different beef and moose treatments (Fig. 5).

The PCA biplot revealed a clear clustering of the unmarinated samples (UB, UM) in the third quadrant of the plot compared to the marinated ones. Moreover, marinated samples are perfectly separated based on the meat type with moose meat (MM, MS) located in the first and second quadrants respectively and beef (BM, BS) in the fourth quadrant. Regarding the sensory attributes, spiciness and sweetness grouped close to marinated moose samples. Volatile terpenes and thiols were observed to have an impact on the organoleptic attributes evaluated in the study shown by the Pearson's correlations obtained between some volatiles and sensory attributes (Table 1). Specifically, Pearson correlation coefficients revealed strong correlations between certain terpenes (e.g. o-cymene, limonene, linalool or tepinen-4-ol) and spiciness and sweetness attributes, while methanethiol was significantly correlated with saltiness and overall preference (*r* = 0.83 and *r* = 0.86 respectively) as shown in Table 1. It is important to note that PCA is an exploratory statistical tool and does not generally allow testing hypotheses. As such, to determine whether there were significant differences in the abundances of volatiles detected in unmarinated and marinated grilled meats, the means of the abundances of the terpenes and thiols detected were compared among the samples using one-way analysis of variance (ANOVA). In line with this, the volatiles dimethyl disulfide and dimethyl trisulfide content, as well as the sensory attribute sourness clustered unmarinated meat samples (UB, UM) in quadrant 3, were significantly higher in unmarinated moose and beef compared to their marinated counterparts (BM, BS, MM and MS). Conversely, the content of the volatiles and sensory attributes scores which grouped marinated moose samples (MM, MS) in quadrants 1 and 2 of the biplot were generally higher in the marinated moose compared to the control moose and beef samples (UM, UB), whereas the abundances of the volatiles endo-borneol, allyl isothiocyanate and 3,4-dimethyl thioene which clustered with marinated beef samples (BM, BS) in quadrant 4 were generally higher in the marinated samples compared to the unmarinated beef (UB), as well as to unmarinated and marinated moose meat samples (UM, MM and MS respectively) as shown in the Supplementary material. See our associated research article for detailed and extended volatile profile and results and discussion [1]. This grouping accounted for 90.47% of the total variance in the data set and has demonstrated to be very useful in interpreting the effect of marinade formulations on volatile terpenes, thiols and consumer sensory perception of grilled meat.

**Table 1:** Pearson's correlations values of the sensory attributes scores and the abundances of the terpenes and Sulphur derivative volatiles detected in unmarinated and marinated grilled ruminant meat.

Number in PCA biplot	Terpenes and Sulphur derivatives	Sweetness	Saltiness	Sourness	Spiciness	Aftertaste	Tenderness	Overall Flavour	Overall Aroma	Overall Preference
1	*α-Pinene*	**0.882**	0.094	−0.188	**0.915**	0.427	0.277	0.472	0.263	0.286
2	*Camphene*	**0.861**	0.057	−0.229	**0.900**	0.403	0.260	0.411	0.223	0.273
3	*o-Cymene or isomer*	**0.888**	0.254	−0.307	**0.967**	0.461	0.171	0.471	0.382	0.454
4	*3-Carene or isomer*	**0.879**	0.083	−0.152	**0.904**	0.437	0.304	0.491	0.254	0.263
5	*α-Myrcene or isomer*	**0.890**	0.169	−0.242	**0.945**	0.446	0.230	0.477	0.318	0.363
6	*α-Terpinene or isomer*	**0.893**	0.144	−0.204	**0.935**	0.445	0.259	0.491	0.305	0.332
7	*o-Cymene isomer*	0.751	0.349	−0.558	**0.909**	0.350	−0.081	0.274	0.394	0.576
8	*Limonene*	**0.889**	0.197	−0.275	**0.954**	0.448	0.204	0.466	0.340	0.399
9	*Linalool*	**0.835**	0.346	−0.459	**0.962**	0.409	0.014	0.386	0.427	0.557
10	*Endo-borneol*	−0.171	0.748	0.170	−0.003	0.239	−0.169	0.518	0.445	0.308
11	*Terpinen-4-ol i*	**0.815**	0.286	−0.465	**0.955**	0.376	0.014	0.367	0.351	0.480
12	*Terpinen-4-ol isomer*	**0.853**	0.304	−0.382	**0.978**	0.418	0.072	0.460	0.380	0.464
13	*Elemene isomer*	**0.867**	0.022	−0.104	**0.870**	0.416	0.339	0.482	0.211	0.200
14	*Carvacrol*	0.740	0.363	−0.585	**0.916**	0.322	−0.124	0.276	0.387	0.566
15	*Carvacrol isomer*	0.754	0.454	−0.546	**0.944**	0.369	−0.122	0.373	0.453	0.602
16	*Carvacrol isomer*	**0.856**	0.404	−0.424	**0.960**	0.463	0.044	0.415	0.509	0.637
17	*Coapene*	**0.879**	0.111	−0.233	**0.925**	0.417	0.240	0.451	0.269	0.309
18	*Copaene isomer*	0.536	0.363	−0.724	0.735	0.186	−0.301	0.034	0.342	0.605
19	*Cariophyllene isomer*	0.529	0.290	−0.726	0.711	0.158	−0.284	−0.020	0.291	0.564
20	*Cariophyllene*	**0.877**	0.075	−0.164	**0.904**	0.422	0.291	0.480	0.247	0.257
21	*α-Guaiene*	**0.860**	0.153	−0.292	**0.939**	0.412	0.188	0.437	0.277	0.340
22	*Humulene*	**0.875**	0.063	−0.135	**0.894**	0.424	0.311	0.492	0.239	0.236
23	*α-Bisabolene*	**0.866**	0.133	−0.235	**0.932**	0.409	0.218	0.478	0.265	0.293
24	*Methanethiol*	0.603	**0.830**	−0.417	0.797	0.481	−0.191	0.516	0.754	**0.858**
25	*Allyl isothiocyanate*	0.333	0.643	0.472	0.111	0.632	0.400	0.538	**0.870**	0.755
26	*Diallyl disulfide*	0.758	0.415	−0.548	**0.908**	0.380	−0.086	0.298	0.464	0.645
27	*Dipropyl disulfide*	**0.814**	0.317	−0.490	**0.934**	0.387	0.000	0.321	0.408	0.564
28	*Allyl trisulfide*	0.770	0.785	−0.181	**0.895**	0.615	0.047	0.748	0.787	0.780
29	*Diallyl sulfide*	0.710	0.164	−0.552	**0.840**	0.288	−0.027	0.148	0.241	0.447
30	*2-Ethyl dithiane*	**0.901**	0.066	−0.143	**0.901**	0.399	0.285	0.489	0.272	0.257
31	*Allyl-2-isopropyldisulfane*	**0.869**	0.234	−0.362	**0.952**	0.432	0.137	0.402	0.364	0.468
32	*(E)−1-(Prop-1-en-yl)−3-propyltrisulfane isomer*	**0.865**	0.139	−0.286	**0.938**	0.397	0.185	0.437	0.271	0.325
33	*3,4-Dimethyl thioene*	0.035	0.667	0.564	−0.110	0.474	0.263	0.562	0.722	0.520
34	*Dimethyl disulfide*	−0.534	**−0.894**	−0.039	−0.696	−0.792	−0.215	**−0.831**	−0.759	−0.754
35	*Dimethyl trisulfide*	−0.494	**−0.906**	−0.010	−0.665	−0.787	−0.191	−0.787	−0.756	−0.777

Values in bold are different from 0 with a significance level alpha=0.05.

The XLSTAT based multivariate statistical approach presented demonstrates an efficient technique useful for elucidating the relationships between volatile metabolites abundances and consumer sensory attributes in grilled marinated and unmarinated beef and moose meats and could be applied to the analysis of other food systems. In the absence of PCA reduction of data sets consisting of qualitative and quantitative variables into clusters based on inherent dissimilarities/variances, rationalizing such data sets would have been a tedious, computationally tasking and slogging endeavor. Thus, multivariate analysis viz principal component analysis (PCA) is an indispensable statistical tool for reducing complex data sets and to better understand the determinants of quality and sensory relationships in grilled food systems.

**Supplementary material and/or Additional information:** Raw data, PCA and ANOVA outputs are available and included with this article as supplementary material.

## Appendix Supplementary materials

Image, application 1
